# The Pathological Histology of Bronchial Affections, Pneumonia, and Fibroid Pneumonia: An Original Investigation

**Published:** 1892-06

**Authors:** 


*The Pathological Histology of Bronchial Affections, Pneumonia, and Fibroid Pneumonia: an Original Investigation.* By A. G. AULD, M.D. Pp. 207. London: J. & A. CHURCHILL. 1891.

This is a valuable contribution to the study of the pathological histology of bronchitis and pneumonia, and will amply repay a careful perusal. It is full of original observations, which are the fruit of much painstaking labour. It is curious that, in spite of the great frequency and importance of bronchitis, observations on the pathological changes in the bronchi are few and inadequate. There will be less reason to complain of this in the future, as the present work in great measure supplies the gap in our literature. The author precedes his descriptions of the pathological histology of the above affections with a short account of the normal structure of the bronchi and lung tissue. Amongst the many interesting features of bronchitis, we may allude to his description of the excessive growth of the glandular epithelium which occurs in the chronic form of the disease. He considers this to be best described as an "atypical epithelial growth," and not as an adenoma. Mention is made of the peculiar band-like formations which traverse the bronchi in cases of bronchiectasis, which are shown to be formed by the growing together of the free ends of papillary outgrowths formed of granulation tissue. On histological grounds the vaso-motor theory of origin of the asthmatic paroxysm is considered to be improbable. Passing on to broncho-pneumonia, the author makes out a fair case for the germination of the fixed connective tissue cells in the alveolar walls, giving rise to a crowd of young cells which pass on into the alveoli, as the typical change in acute broncho-pneumonia, in opposition to the general view that it consists of proliferation of the alveolar epithelium. The latter change, he says, may occur in the chronic form of the disease, but is not characteristic even of this.

A good account is given of the minute changes in the lung in acute pneumonia. When resolution is delayed, it is shown that there are morbid changes in the alveolar epithelium. The author endeavours to prove, from a careful examination of several cases, that a chronic induration—fibroid change—may arise out of acute pneumonia; the intra-alveolar fibrinous exudation first undergoing fibroid organisation, and the adventitious tissues being secondarily affected; and that, in the second place, there is an affection of the lungs anatomically commencing in the same way as the above—namely, by induration of a creeping fibrinous exudation, by interstitial changes and epithelial proliferation and degeneration, but arising independently of acute pneumonia—this he calls "fibroid pneumonia." In their late stages the profound alterations produced in the lungs probably render these affections indistinguishable from other forms of pulmonary cirrhosis.

The book deserves careful study; and some well-executed drawings of the microscopic appearances, which are given, are a considerable aid to the clear understanding of the descriptions in the text. The book is clearly printed. We do not like to find fault with a work of real merit, and one which we hope will meet with the success that it deserves, but we must say that it is a pity that more care was not taken in the revision of the proof-sheets. Some of the sentences are involved and obscure in meaning, there are many slips in spelling and some in grammar, and other signs of careless revision; for instance, one writer's name is spelt in two different ways on the same page, and in a reference to one of the author's own papers in the *Lancet* the date is given as 1861 instead of 1891. We can recommend the book to our readers, and no doubt in future editions these small errors will be corrected.

